# Working with parents of children with complex mental health issues to improve care: A qualitative inquiry

**DOI:** 10.1177/13674935211028694

**Published:** 2021-06-28

**Authors:** Brenda MY Leung, Cynthia Wandler, Tamara Pringsheim, Maria J Santana

**Affiliations:** 1 Faculty of Health Sciences, 4512 University of Lethbridge, Lethbridge, AB Canada; 2 Morinville, AB Canada; 3 Department of Clinical Neurosciences, Psychiatry, Pediatrics and Community Health Sciences, Cumming School of Medicine, 70401 University of Calgary, Calgary, AB Canada; 4 Departments of Paediatrics and Community Health Sciences, Cumming School of Medicine, 70401 University of Calgary, Calgary, AB Canada

**Keywords:** Child and adolescent mental health, health services research, patient-centred care, qualitative research

## Abstract

The study objective was to explore the experiences of parents of children (6–17 years) with complex mental healthcare needs in accessing healthcare services in Alberta, Canada. Parents were interviewed using a semi-structured guide with open-ended and probing questions. Interviews were audio recorded and transcribed verbatim. Thematic analysis revealed three main themes: (1) *Fragmented healthcare services* profoundly impacted participants’ experience of mental health care due to (a) a lack of a collaborative approach across disciplines in the healthcare system; (b) unavailability of information related to mental health care and (c) a lack of patient-centred care. (2) *Navigating the complex healthcare system* was difficult due to fragmented services and was hindered by gaps in accessing and receiving care, lack of continuity of care and lack of resources. (3) *Distressed parents* discussed the emotional challenges, financial burdens, self-advocacy and stigma they experienced in navigating the system. Parents offered insights into potential solutions to these gaps. Parents recommended the creation of a one-stop shop service with a team approach led by a navigator to facilitate and support navigations across healthcare services that work collaboratively across disciplines among healthcare services and across sectors inclusive of social services, education, policing and community programmes.

## Introduction

Families of children with complex mental health care needs face significant challenges in the search for timely and appropriate services (Sayal, 2006). Issues include contacting multiple agencies, looking for multiple types of treatments and help for different problems across agencies, resulting in increased burden on the mental healthcare system and the families (Reid et al., 2011; Schraeder and Reid, 2015). Moreover, the process of seeking help by families has been described as ‘circuitous and long’ and the pathway for obtaining a diagnosis for their children is often ‘confusing, distressing and uncertain’ (Clarke, 2012). The help-seeking process can leave families without appropriate access to services and treatment to support their child’s mental health (Clarke, 2012).

About 20% of Canadian youth experience mental disorders (Comeau et al., 2019) with the prevalence steadily increasing (Leung et al., 2019). Children and youth mental healthcare services are provided in the community via clinics, private practice, hospitals and schools (Canadian Institute for Health Information, 2015). Well-recognized gaps in the care delivery system include service availability and integration, timely access and transition of care from youth to adulthood (Canadian Institute for Health Information, 2015). In fact, identifying challenges in service provision to address unmet needs have been identified as a priority for researchers, policymakers and administrators (Duncan et al., 2020).

Experience of families with children living with complex mental health problems remains largely unexplored (Carman and Workman, 2017). Anecdotal discussions with parents reveal a lack of treatment programmes, a lack of information about existing programmes, poor communication between healthcare departments, lack of information about medication safety and efficacy and a lack of adequate treatment monitoring (Clarke, 2012). While we understand the sociodemographic characteristics of those who struggle to find services, such as severity of mental disorder, parental perceptions, child age and gender, and family and social background (Sayal, 2006), and the reliance of current practices on short-term reactive interventions (Reid et al., 2011), less is known about the struggle of families dealing with limited options for treatment and a lack of support for accessing services. Only recently have issues of stigma experienced by families been contemplated; that is, stigma is important as a driver of trauma and emotional burden for the families (Hinshaw, 2005).

Currently, little is known about challenges that families face in their search for resources and navigating the healthcare system to find options for their children. In this patient-oriented research, voices of families are captured to build research evidence to improve access to healthcare services and policies.

### Aim

The aim of this study was to explore experiences of parents of children with complex mental healthcare needs in their journey across healthcare services in Alberta, Canada. Specific objectives include identifying gaps in care and proposing recommendations to improve care.

## Methods

This patient-oriented qualitative study conducted one-on-one semi-structured interviews with parents of children living with complex mental healthcare needs. According to the Canadian Institutes for Health Research-Strategic for Patient-Oriented Research definition, the term patient refers to individuals with personal experiences of a health issue and/or informal caregivers, including family and friends (Canadian Institutes of Health Research, 2019). An advisory group of three parents from rural and urban areas worked with the research team to advise and support the study to completion. This advisory group co-designed the interview guide with the research team and supported the members’ checking activity as well as the final writing of the manuscript. The interviews were conducted face to face, by video, or by phone, to accommodate the preference of the participants. We conducted thematic analysis using an inductive approach.

### Inclusion criteria

Parents met inclusion criteria if they had a child aged 17 or younger at the time of accessing healthcare services in Alberta, diagnosed with depression, mania (hypomania), anxiety or bipolar disorder and were fluent with written and spoken English. Potential participants contacted the research team and were screened by telephone. Those who met criteria were invited to participate. Parents who consented were interviewed by one of the investigators [BL, TP or MS].

### Recruitment

Diverse strategies were used to recruit participants. Parents involved in a previous study (Leung et al., 2018) were invited to participate. These parents contacted other parents through their networks, and then snowball sampling continued. In addition, other recruitment strategies included posting information about the study in medical clinics (family doctors, paediatricians and psychiatrists/psychologists), shared with community partners (such as the Family Centre, Addictions and Mental Health Strategic Clinical Network® and Alberta SPOR Support Unit), and through social media such as Facebook posts.

### Data collection

A semi-structured guide with open-ended questions and probing questions was used (see Supplementary information Appendix A – Interview Questions for semi-structured interview). Interview questions were piloted to assess for language (understandability), relevance (i.e. makes sense to patients) and comprehension (i.e. content validity). Pilot testing of the interview questions was performed by three parents not in the study, who reviewed the questions and provided feedback on language, relevance and comprehension. Feedback from parents was incorporated to revise the interview guide. Data were collected to saturation where no new themes were derived from the data. Interviews were conducted from October 2017 to April 2018.

A questionnaire collected information on demographics of (1) the child: gender, birth date, grade, type of school, diagnosis, medication and supplements and (2) the parent: gender, age group, marital status, education and annual household income (Table 1).

**Table 1.** Demographic and health-related characteristics of parents and their children.

Characteristic		Frequency (%)
Parents		
Marital status	Married/common law Divorced/separated	13 (81%) 3 (19%)
Education	Technical/professional college University or higher High school/some college	6 (37%) 7 (44%) 3 (19%)
Household income	$15,001–$30,000 $30,001–$60,000 $60,001–$80,000 $80,001 or more	3 (19%) 2 (12%) 3 (19%) 8 (50%)
Urban/rural	Urban Rural	12 (75%) 4 (25%)
Family mental health history^a^	Major depression Anxiety disorder Problems with alcohol Manic depression/bipolar disorder Behaviour Problems ADHD Learning problems Problems with illegal drugs Problems with legal drugs Schizophrenia	14 (87%) 13 (81%) 10 (62%) 7 (44%) 7 (44%) 7 (44%) 7 (44%) 6 (37%) 4 (25%) 1 (6%)
Children		
Gender	Male Female Other (non-binary)	7 (44%) 8 (50%) 1 (6%)
Age group	9 and younger 10–16 17 and older	3 (19%) 9 (56%) 4 (25%)
Number of diagnoses	1 diagnosis 2 diagnoses 3 diagnoses 4 diagnoses 5 diagnoses	2 (13%) 1 (6%) 7 (44%) 5 (31%) 1 (6%)
Diagnoses^a^	Anxiety disorders Attention deficit hyperactivity disorder Depression Obsessive compulsive disorder	14 (88%) 8 (50%) 6 (38%) 5 (31%)
Physical health status	Poor health Fair Good Very good Excellent	1 (6%) 1 (6%) 2 (12%) 6 (38%) 6 (38%)
Mental health status	Poor Fair Good	8 (50%) 7 (44%) 1 (6%)

^a^Participants checked or listed multiple options to questions on family mental health history, child’s medication and supplement use; hence, total adds to >100%.

### Data analysis

Qualitative data collected via interviews were audio recorded and transcribed verbatim, with anonymized names and locations. Thematic analysis of text data was undertaken by two researchers independently. A total of four team members coded the data, and two researchers developed the themes in an iterative process with constant check-ins and comparisons to arrive at consensus.

The researcher read each transcript several times to become familiar with the data. Then we followed the inductive approach of thematic analysis (Braun and Clarke, 2014; Maguire and Delahunt, 2017) to identify the initial codes from the data, using a line-by-line coding. The coding was data driven, meaning the themes were extracted as the data unfolded, using open coding (i.e. not relying on pre-set codes). Each segment of relevant data was coded to produce as many patterns as possible. Each transcript was coded separately by at least two analysts and codes were reviewed, compared and refined to arrive at a consensus. Once the coding was completed, the codes were organized to identify themes.

Themes were derived from the coded data. Emerging themes were obtained with descriptive categories and subthemes (Morse, 2008). Thematic analysis commenced at the beginning of data collection, where researchers familiarized themselves with the data and generated initial ideas of themes to explore in subsequent interviews. Due to this iterative and reflective process, researchers were able to identify the point at which data saturation was reached. Differences with respect to emerged categories or themes were resolved by discussion and consensus by the research team. Overarching themes were formulated to provide insight into the research questions.

The advisory group reviewed transcripts and the themes derived from the data for validity check. Additionally, we used the Consolidated Criteria for Reporting Qualitative Research checklist (Tong et al., 2007) to report all aspects of the study including analysis of the data and interpretation of the results.

Quantitative data from the demographic questionnaire were used to describe the sample characteristics using frequency, proportion, mean and standard deviation (where appropriate).

## Results

A total of 36 parents expressed interest in participating and were invited to participate in the study. Of the 36 parents, 16 (15 mothers and one father) were interviewed. Interviews lasted 60–90 min. The reasons for individuals not interviewed included not returning calls/emails, *n* = 8 (22%), incomplete screening, *n* = 5 (14%), loss of interest, *n* = 3 (8%), and not providing consent, *n* = 3 (8%).

### Characteristics of participants

Characteristics of the parents and children are presented in Table 1. Majority of parents were married, *n* = 13 (81%), had college/professional or university education, *n* = 13 (81%), and had household incomes over $80,000 Canadian, *n* = 8 (50%).

Children were equally distributed by gender, with one child self-reported as non-binary. The largest age group was 10–16, *n* = 9 (56%). For the child’s physical health status, a majority, *n* = 14 (87%), reported being in ‘good’, ‘very good’ or ‘excellent’ physical health. For mental health status, *n* = 8 (50%) reported having ‘poor’ status and *n* = 7 (44%) experienced ‘fair’ mental health. Of the 16 diagnoses reported by parents, the majority of children, *n* = 14 (88%), had two or more diagnoses. The most common diagnoses among the children were anxiety disorders, *n* = 14 (88%), followed by attention deficit hyperactivity disorder (ADHD), *n* = 8 (50%), depression, *n* = 6 (38%), and obsessive compulsive disorder, *n* = 5 (31%).

Parents identified a total of 13 medications prescribed to or used by children for their psychiatric condition. The most common drugs used were antipsychotics (risperidone and aripiprazole), antidepressants (fluoxetine and sertraline) and ADHD medications (methylphenidate and guanfacine). Of the drugs used, six children were on antipsychotics, five children used ADHD medications and nine children were on antidepressants. One child used medical marijuana, and one child did not use any medication.

Parents reported the most common supplements given to children were vitamins, *n* = 11 (69%), followed by fish oils, *n* = 4 (25%), probiotics, *n* = 3 (19%), and melatonin, *n* = 3 (19%). Many children took more than one category of supplements. Four participants, *n* = 4 (25%), reported no supplementation use. Parents reported multiple psychiatric conditions in their family history, with the most common being anxiety and depression, *n* = 13 (81%) and *n* = 14 (87%), respectively, and alcohol use, *n* = 10 (62%), with one family reported a family history of schizophrenia.

### Themes derived from the data

Analysis of the data generated 96 different labels that were then assigned to three main themes: (1) fragmented healthcare services, (2) navigating the complex healthcare system and (3) parents distressed by the system. The following section presents the themes and subthemes (i.e. the gaps identified) and the solutions proposed by the parents.

#### Theme 1: Fragmented healthcare services

Fragmented healthcare services profoundly impacted participants’ experience of mental health care. The first theme is further supported by three subthemes:

##### Subtheme: Lack of collaborative approach across disciplines including school and community services

Parents reported a lack of collaboration and communication among healthcare professionals in different departments, schools and the community. The services provided by different professionals such as school teachers and counsellors, or sectors such as community services and clinics, are fragmented and disjointed without clear communication among them.

##### Subtheme: Unavailability of information related to mental healthcare services

Information about resources and treatments was hard to find. Parents were occasionally provided with misinformation. Parents highlighted the lack of resources specifically related to the lack of knowledge by educational and healthcare professionals about available resources and programmes for supporting mental health and education.

##### Subtheme: Lack of patient-centred care approach

Parents described gaps in patient-centred care, specifically highlighting the need for compassionate care and engagement in decision-making. Parents’ input was not considered when deciding on a treatment plan. Thus, parents felt sidelined in deciding how best to address child’s needs.

Table 2 displays the categories within the subthemes and their respective quotes from participants about their experiences.

**Table 2.** Theme 1 subthemes and quotes from participants.

Theme 1: Fragmented services		
Subthemes	Category	*Quotes*
Lack of collaborative approach across disciplines	Communication	*“… we’ve got the doctor, we’ve got the pediatrician, we’ve got the Mental Health, we’ve got FSCD [Family Support for Children with Disabilities], we’ve got all of these different pieces, they’re all working beautifully on their own and none of them are working together and none of them has any clue what the rest of them do. And I think in our time experience that has been, across the board, this is not isolated to healthcare.” E003*
	Collaboration among service providers	*“…I get told quite often by my kids at school, that what my like-, our psychiatrist recommends is not available, because the school district simply either doesn’t do it anymore, doesn’t have funding to do it uhm, …… they don’t have the people to-, to do the testing, that kind of stuff. So, uhm but the doctor is like “well, you should just be able to go to the school district and ask for it” L014*
Unavailability of information related to mental healthcare services	Information about resources/programs	*“Nobody told us about [a program supporting mental health care], I found [this program] on my own. And I was really surprised that with the issues we were having, nobody told us to contact them … nobody had said, you know, we think you would benefit from this program…” E004*
	Information about medication and treatment options	*“I said I talked to four healthcare professionals to know about the affective mood disorders clinic. Every single one of them has told me something different and I said now I find out that the clinic isn’t even offering, it is closed, and she said to me who told you that. I said the pediatrician, she said no, she said the clinic operates. She said I’m directly affiliated with it. It took me three or four months just to get that information.” L011*
	Parents’ reliance on the Internet to find information	*“with our issues and [child’s] age and needs, nobody told us to contact [program name] … I did a lot of Googling ….” L002*
		*“The concerns are just with regards to getting enough information, you know, is it really gonna be helpful? Enough information regarding how the two drugs would interact with each other… Yeah. No, we have to Google that ourselves. Very unfortunate.” E004*
Lack of patient-centred approach	Compassion care	“…..*the care is meant to serve the system rather than a patient*” L003
		*“She said, “what do you want”, I said, “I want those [behaviours] to stop” and so he did but then his grades plummeted because he was now not getting rid of the panic before school… …And so, I said to her, you know his grades have gone down…. not doing well in class, … getting in trouble…. And she’s like well, you told him that you didn’t want to be yelled and screamed at in the morning anymore, he’s not yelling and screaming at you anymore… You’re asking for a moving target… And I said no “I’m asking for us to get some help so that he doesn’t have, he can deal with his anxiety better because yelling and screaming at me is not the way to do it, …and acting out in school now, is not the way to do it…” but she closed the file because we had the goal when we went in for our 10 sessions, was to stop the outbursts on going to school… We had completed… we had reached our goal.”* E015
	Engaging parents in decision-making and treatment plans	“*we couldn’t talk directly with the psychiatrist; we had to do it indirectly through the psychologist at [public funded system]*” E011
		*“…. I’ve been doing this for 10 years and then I go to somebody here and they act like I don’t know anything and when I say my child needs this, they are like – well, no, we’re going to hold off. And then it puts my son … where he is emotionally in a panic mode. He doesn’t know what to do with his health. He, he’s just losing it, and it’s scaring him. And I can’t help him.”* L02

#### Theme 2: Navigating the complex healthcare system

Navigating the complex healthcare system was hindered by gaps in accessing and receiving care, lack of continuity of care and lack of resources. The inability of parents to find and access appropriate services led to increased visits to the emergency department. These gaps were systemic, such as a bureaucratic structure making referrals a complex process or inappropriate information (including referrals) provided by clinical staff.

Table 3 displays the categories within the subthemes and their respective quotes from participants about their experiences.

**Table 3.** Theme 2 subthemes and quotes from participants.

Theme 2: Navigation of complex healthcare systems		
Subthemes	Category	*Quotes*
Barriers to accessing and receiving care	Geographic location	*“… we drove a lot, right? We’re in [Town 1] , so we drove there once a week, we were in [Town 2] once a week for therapy, and then we were in [City 1] for psychiatrist, and then we were in [City 2] for neurologist. So, we travelled, there were a lot of miles and not much sleep in that time and it definitely impacted our family…….it definitely, that was probably the toughest thing, was just with no home….It was constant and exhausting because you had to travel for everything, right?”* L012
		*“Yes, well… what’s the longest distance we’ve had to travel? We had to go to [City 1] and we had to go to [City 2], but I mean again… Uhm, it’s, what’s that word? Reimbursed! Or covered in some manner, right? They are pretty good about that but that’s through income support. So, if I wasn’t on income support, I mean, I wouldn’t have that, right? I wouldn’t have that, that care.” E008*
	Referral and long wait times	*“to see a psychologist at the hospital in a rural community it took a year of trying for the new person in her school to refer to one of the hospital" E013*
		*“So, I’m really grateful, that we got into a medical professional who was able to diagnose properly and medicate, I’m sad that it took us 12 years to get there.” E015*
		*“... when he, my son, told me that he was going to kill himself, I phoned in and was like “hey, so, what is the protocol for this?” and they had me booked in within... within two weeks.” E016*
		*We’re waiting on a feeding and swallowing clinic; she has an appointment, but that referral took us, to get that referral took us three months of fighting to get that referral. And then from the referral till the time they called us, it was about three weeks and then our appointment was another, six weeks?“ E004*
		*“I went to our general practitioner, and then he referred us to Children’s Mental Health. So, initially our wait list for Children’s Mental Health was 8 months…” E016*
	Lack of continuity of care	*“I have my own three binders that I bring to every appointment because the continuity of care is not there. It’s all very confusing and there are so many things that you could access but you really don’t know how to do it, unless you know where to look” E007*
		*“they are passing the buck, where everybody is like – yes, I see she has an issue, however, I don’t want to deal with it, let’s send her here. And then we go there, and then they’re like - oh well, we see there is an issue, but we’d best feel you would be seeing here…” E004*
	Lack of timely and proper diagnosis	*“I don’t think she has actually said that DDD has anxiety or whatever, depression, but, umm…she knows she needs help… I know she knows, she has said she needs, she help with her problems. But, so far there’s no specific diagnosis from the psychologist about what she had.”* E013
		*“So, I’m really grateful, that we got into a medical professional who was able to diagnose properly and medicate, I’m sad that it took us 12 years to get there.” E015*
	Treatment plan and follow-up	*“My son has been in many-many clinics and so we’ll see a doctor for a short period of time and we’ll get a referral to a certain doctor... And generally, we see them about three times before they decide that he is too complex for their office. And then they will refer him on to another office, in which time I have no, no follow-up, nobody to monitor him, nobody to call when there is a problem. And I have no timeline on when I’ll see that person. Then when I do get the appointment with that person it’s the same thing again. Generally, they see him two to three times and then they move him on.”* E007
		***“****… there’s no continual care whatsoever for him, from him. As like – oh, you’re fine for six months, okay, well sorry, see ya! You know, well, and especially with mental health, mental health is something that can be really good for a period and then really bad for a period. It’s not something that you can just fix like a rash or an illness that requires antibiotics, right, exactly! It, it may kind of go to sleep for a little while, but it doesn’t mean that it's gone.”* E012
		*“So, that was a 10-week program. So, by then, so that started in January and about five weeks in [child’s] behaviour was going off the rails. So, if it had been up here before now it was up here, and I started meeting with the lead psychologist for the group therapy and I said to him you know it is a great program, but it ends in five weeks. I need to put other supports in place for my child so that when it’s over she has somewhere else to go.”* E011
		*“We knew we were probably another year out from seeing a psychologist, but we’d had no support for 16 months right?”* E015
Lack of resources at community level		*“There haven’t been any real uhm, like any sources that I can find anyway that are covered by health care… any real like, counselling with a, a qualified psychologist or counsellor.” L014*	
		***“****It’s not working at all. Like I mean, it’s very based on school jurisdictions, sadly. Uhm… it is very broken, I know for [child], he is still going through a mental breakdown right now; I can’t get him to school. Uhm… we have a doctor’s note from the psychiatrist, basically that says until the end of June [child] is unable to cope because of developmental trauma. And it’s pretty sad because education is a basic need, he’s far behind and I have to pay for a tutor. And he’s so stressed out at school that he misbehaves, and then he keeps getting punished. So, it’s… the education system has re-traumatized an already traumatized child. It’s what it’s done for him.”* E010	
		*“It’s been an absolute nightmare. He has suffered so much. The first year we moved here, he came home from school every day in tears just bawling and it’s because he, he lacked that, that support and there was no connection between the health aspect and education aspect. And I mean let’s face it, if you’re not healthy, how can you go to school? Whether it’s mental health, physical health, whatever. You are not gonna gain any knowledge, right? So, I really think that there is absolutely no connection here whatsoever and there should be.”* E012	

### Subtheme: Barriers to navigating and accessing care

Barriers disclosed by the participants included difficulties in accessing care due to:

i. Geographic location: Parents living in rural areas had a hard time accessing services as most services were located in urban centres. Driving time as well as indirect and direct expenses attached to driving to urban areas or to bigger rural areas presented a barrier to accessing and receiving care.
ii. Lack of continuity of care: Parents described the gaps related to care that is not well-organized as being affected by slow referrals to specialist care and long wait times, lack of proper and timely diagnoses and lack of treatment plans.
iii. Referrals and long wait times: Parents waited for referrals to specialists, ranging from school to counsellor, from family doctor to paediatrician, psychiatrist and also to psychologists.
iv. Lack of timely and proper diagnosis: Families often required a diagnosis for their child to receive treatment or referrals to programmes. However, obtaining a diagnosis was often fraught with delays and inadequate assessments.
v. Treatment plan and follow-up were insufficient as services were often provided within time frames that did not meet the needs of the child.

#### Theme 3: Distressed parents

Distressed parents discussed the struggles and suffering that parents and families with children with mental health problems face when trying to understand a fragmented system that is hazardous to navigate. The struggles are related to emotional challenges, financial burden, self-advocacy and stigma. Table 4 displays categories within the subthemes and their respective quotes from participants about their experiences.

**Table 4.** Theme 3 subthemes and quotes from participants.

Theme 3: Distressed parents	
Subthemes	*Quote*
Emotional challenges	***“****There were days when I feel like I need to go to the Mental Health cause I’m so frustrated. Yeah, it kind of filters through the whole family then when your kid is you know exploding and you’re not, she is not receiving care and instead of me focusing my energies on helping her I’m focused on trying to navigate a system that doesn’t work.”* E003
	***“T****he ups and downs of having a kid with special needs is, is complex. I mean it’s very difficult to deal with those ups and downs. But when you add the middle times when you don’t have care, when you don’t have somebody to follow-up with it makes it a lot more stressful for the parent. And you don’t know what to do. And that anxiety passes on through the kids; and they can feel it and they’re, you know, more anxious; and then you’re going – oh my goodness, what am I doing?!” E007*
Financial burden	*“So we did testing, we paid for that, we also pay for all the psychologists… Out of pocket… Well, some benefit coverage but then out of pocket, because it doesn’t cover enough, right? ... and then we thought we’d pay for a psycho ed assessment.”* E004
	***“****I’ve been on an unpaid leave from work for a year, it will be a year in May, I saw the first time, like I had to take a leave for 3rd time and so, you know, we have these additional financial strains in terms of therapy and in the natural path and stuff and less money coming in because I don’t work, because I have to support this child, support our family, so you know what about the single-parent families and stuff?, It’s awful.”* L011
	***“****It’s challenging to have a child with special needs and be a full-time worker. I say all the time, probably daily I have to make a decision of whether I’m going to be the best parent or the best employee. And that’s a, that’s a decision nobody wants to have to make. I don’t want to choose my work over my kids. But there’s moments where you, you need to be at work and, and you can’t be there for your kids.”* E002
	***“****I have three kids and I can’t take all three kids to an appointment. So, it has resulted in my husband having to take a lot of time off work to kind of coordinate that care. Because when you have all of these different things working together we also were not in a position where we could just drop our kids off anywhere. Right now. And so I don’t what the solution to that might be, but it would, it’s extended hours at clinics would be helpful or even if there was an area that I know my kids are safe while I’m in my appointment or something, something to coordinate the fact that I have more than one child.”* L003
	***“****Being a mom who has three kids with special needs and all go to so many different places and being on income support, uhm, the transportation issue has really been a stressor because gas is expensive. Especially nowadays, it’s like on sky high and so having to, I mean I’m not trying to say that is not my responsibility, it’s totally my responsibility but one thing that has been nice is with income support after having the support letters written, they do reimburse for some gas. So that has been good.”* E012
Parents must self-advocate	***“****Lots of notetaking. When I walk into my appointment, I’m always very strategic, where I have certain highlights that I need to fit. I focus on those first, and then we deal with whatever agenda the psychiatrist may have or the psychologist may have. Perfect example was a while back that psychologist worried that [child] was getting depressed. So I, the onus was on me for my appointment with the psychiatrist that happens to be a month later to essentially request that he does the depression assessment, I think it was called? And he did it and then the onus was on me to go back to the psychologist to provide with the results and I’m not an expert. I know what I know from reading, which, you know, every article that’s written out there has a different opinion, so I could be missing…int... uhm misinterpreting what they’re saying… I could be, which is a bit scary cause that affects [child] too, right?”* E010
	*“And I said put her on a waiting list, please. They said we don’t have such a thing. I said – make one. I said I work in a healthcare system, I get short-notice cancellations all the time, put her on that list, make one. And I was very tight about it, and she made one for me…yeah but she, I mean, the list doesn’t exist, but she made one for me because I asked for…we had to do a lot on our own, yeah, a lot.”* E002
	*“He was really surprised I wanted to go immediately into the medication, he was like “wow, most parents are just like ‘you know let’s try...” and I’m like - if my child had cancer, I wouldn’t say let’s give it a month, and see how he does on his own - I would say okay, let’s start treatment… So, he gave me medication uhm, he started [child] on Prozac and Concerta right away.”* E016
Stigma	“*They look at it as a, you know, something’s wrong with you or you’re a bad parent that’s why your kid is not behaving well and is having issues…and so there’s tends to be sometimes blame and judgement…by the doctor rather than listening and referring to a proper source*” E001
	*“at the end of the day and that’s what you tell yourself an awful lot when everyone, when everyone in the world, is judging you, right? That’s exactly what you tell yourself, right? So…a massive amount of judgement!”* E004

##### Emotional challenges

Parents described their struggles and trauma related to a fragmented system that is difficult to navigate. Their struggles affect not only their children, many of whom are dealing with suicidal ideations and violent behaviours, but also the family at large, specifically stressors on marriages, single parents and other siblings, in addition to the time commitment involved around the parents’ other daily responsibilities.

##### Financial burden

Parents faced a considerable financial burden when paying out-of-pocket for services that are not funded to avoid long waits, when treatment did not work, and when their child’s needs were not addressed. Financial burden adds stress to parents that are already struggling to care for their child with the mental health disorder, as well as their other children.

##### Parents must self-advocate

Navigation within the fragmented healthcare system is impeded by poor continuity of care, and parents need to become advocates for their children needs. Some parents took notes during their visits and others informed themselves using Google to discuss their children care plans, including medications.

##### Stigma

Stigma came from healthcare providers, friends and family and educators at school. Parents felt blamed, judged and socially isolated because of their child’s behaviour. Parents felt they were judged as ‘bad parents’ for their child’s behaviour instead of receiving the compassion and understanding they needed.

## Proposing solutions

Parents proposed solutions when they answered the open-ended question about their vision of ‘an ideal world of a healthcare system that works to meet the needs of both you and your child’. Parents highlighted a need for well-*coordinated care* across the continuum based on a team approach, that includes navigators and enhance continuity of care to address the existing gaps and promote the integration of healthcare services.

A *team approach* is described by the parents as healthcare teams where providers work collaboratively to share information, communicate directly with each other and parents and co-design treatment plans for their child’s care. Parents suggested a central location, a one-stop shop including a multi-disciplinary team with the active involvement of a physician trained in mental health, would more effectively address the disintegration of existing services already in place.

*“to have a team of people that work together in a central location would be valuable or even to take it a step further to have a psychiatrist in that role might be really valuable as well…and I think that when you have a three-year-old on psychiatric medication, the next natural step would be have a psychiatrist to oversee that” [L003]*

Parents suggested centralized coordination with a navigator to oversee the team and its functions in the child’s overall care. This would mitigate many deficiencies in the system, including frontline staff/professionals (including doctors) who lack knowledge of the resources available and are unable to direct families to those resources efficiently.

The *navigator* could be a case manager or resource worker to assist children with complex medical and mental healthcare needs to follow through with all the different specialities and be the one contact for all aspect of care needed by the child.

*“that worker would know ….. [the] issue, then [recommend] this will be the route to go and I’ll make that initial phone call for you and I’ll initiate the referral or – here’s the phone number for you”. Instead, currently, a parent stated “I have my own 3 binders that I bring to every appointment” because the continuity of care is not there. “It’s all very confusing and there are so many things that you could access but you really don't know how to do it, unless you know where to look”. [C007]*

*“Mental health counsellor – like at Key Connections, e.g. monitor disability (claim) for the insurance company – be the liaison between all of those people to make sure that everybody has the same information (parents, doctor, school)” [C007]*

Establishing a team approach with centralized coordination of care would enable streamlining and the continuity of care. In addition, this *streamlined approach* would also allow for early interventions before the child’s condition deteriorates, requiring more intensive and extensive care.

*“Don’t wait until things are dire, which is a level of service that’s incredibly expensive; behaviours get so bad to the point that’s ... hugely stressful and potentially very dangerous for the, for kids and families to be, to be in positions where they are having to kind of navigate this on their own”; “need to catch it earlier, need to recognize the complexity (of what these children need)”. [C006]*

Parents also cited the need to have *cross-sectoral cooperation* of services and professionals in the areas of health, social services, policing and education, at minimum having a line of communication between sectors/professionals:

*“parents [are] willing to sign a waiver so that communication between services, departments, professionals, etc. can take place”. [L003]*

Another parent proposed a triangle of home–school–community working together:

*“they [can] hand you a package and be like this is what’s available to you, like this is what Child’s and Family Services does, this is what Child’s Intervention does, this is what Mental Health does”. [L003]*

Parents living in rural areas suggested the use of videoconferencing, so therapists, practitioners and other professionals working with the child could be ‘on the same page’, having an open-line of communication and shared knowledge of various aspects to the child’s care.

## Discussion

This qualitative study heard from parents of children with complex mental healthcare needs and learned about their experiences in searching for and accessing mental healthcare services in Alberta, Canada. Parents in our study came from diverse sociodemographic backgrounds, yet their shared experience of accessing care for their child was similar. That is, accessing and navigating the system was difficult regardless of education, income or urban/rural residency. For example, a parent clearly described their struggles to access care living in a rural area, despite having higher education and socioeconomic background that was similar to another parent living in an urban centre but had lower income. Parents highlighted a number of gaps and areas needing improvement. Parents offered insights into potential solutions to these gaps.

In many ways, our results are neither new nor startling but confirm the experiences of families and their concerns and frustrations with regard to the functionality of the healthcare system and its treatment of patients with mental health problems (Boulter and Rickwood, 2013; Crouch et al., 2019; Reardon et al., 2017). Our findings are echoed in a report to The United Way–Calgary and Area which also indicated the inability to access mental health services and supports (i.e. ‘Getting In’ to the system), lack of assessments and support for the whole family, the lack of supports while waiting for and beyond ‘treatment’ and transitioning from adolescent mental services to adult services (German et al., 2018). Their findings also suggested a lack of integration across government, non-government and private organizations at the provincial level. The experiences are similar in both Alberta and British Columbia whereby families had difficulties in accessing screening, diagnostic and interventional services because of geographic isolation, transportation limitations, costs due to limited numbers of healthcare providers responsible for widespread service delivery to a broad geographic catchment area and barriers to services when transitioning from one age group to the next (Young et al., 2019).

### The shared experience of families

The challenges experienced by parents in our study are not unique to the Canadian context. The literature highlights parents from a number of Western countries also facing challenges in navigating complex systems, through an often arduous process, to obtain appropriate mental health care for their children (Bone et al., 2015; Boulter and Rickwood, 2013). Parents found it difficult to understand the process to obtain help and encountered numerous obstacles.

Similar to our findings, studies also highlighted parents having to deal with stigma, the lack of integrated healthcare services and a shortage of providers with the expertise in early childhood mental healthcare (Walter et al., 2019). The literature on stigma and mental illness is large; often, stigma experienced by children and their families lead to shame and low expectations, which cause further distress (Heflinger and Hinshaw, 2010). Stigma is a barrier to parents’ help-seeking behaviour, in particular where parents felt ‘blamed’ by professionals (Reardon et al., 2017). Strategies to address stigma need to be done with community, public and social policies as well as health care to shift it (Hinshaw, 2005). The barriers to accessing services can occur in various stages of the help-seeking process, from parental attitudes that influence help-seeking behaviour to accessibility that influences ability to contact services (Reardon et al., 2017; Reid et al., 2011). Conversely, access to services is more likely facilitated by factors such as having affordable and flexible support, enabling trust to be developed with practitioners and reducing stigma for parents and providing clear information on how to access services (Reardon et al., 2017).

Parents expressed a lack of confidence in their healthcare provider’s expertise; they were also unsatisfied with the monitoring of their children’s medication use (Lake et al., 2015). Similar to parents in our study who had to ‘self-advocate’ for care, parents often play a navigator role in accessing mental health treatment and the use of specialist mental health services, thus putting the burden on parents to recognize their child’s mental health needs and having to figure out a pathway for obtaining appropriate care (Crouch et al., 2019). Thus, from Canada to the United States to Australia, families are emphasizing the need for supporting parents in their critical role of identifying mental health problems in their children and gaining early access to appropriate mental health care (Boulter and Rickwood, 2013).

### Parents’ vision for ideal care

Parents recommended the creation of a one-stop shop for services with a team approach led by a navigator to facilitate and support coordinated care across disciplines and sectors, from health care to schools, social service, among other community services. Care coordination has a positive effect on timeliness of care (Miller, 2014), including rapid access and early interventions (Settipani et al., 2019), and promotes favourable attitudes by the care team for the children in their care (Young et al., 2020). Coordinated care has been reported to be desired by parents and beneficial for children with mental healthcare needs (Miller, 2014; Settipani et al., 2019); however, it remains an unmet need with a number of barriers to its implementation (Brown et al., 2014). These include lack of service knowledge, limited time and communications (Young et al., 2020), as well as lack of coordination across sectors leading to discontinuity of care and reliance of parents to act in a liaison capacity between practitioners and sectors (Tobon et al., 2015).

Coordination of care across sectors is especially challenging for children with complex mental healthcare needs (Tobon et al., 2015). Barriers to cross-sector collaborations are often due to ideological and structural differences (Wiart et al., 2010), as well as ineffective coordination of services between systems and lack of mutual understanding (Mikkelsen et al., 2013). A number of facilitators have been proposed to overcome these barriers. For example, leadership in the respective organizations can promote a shared culture of care, long-term trusting relationships and key players as ‘bridge builders’ to build knowledge, attitudes and skills for sharing a common vision and language with all partners (Martsolf et al., 2018). Thus, a multi-faceted approach is required to streamline procedural differences and increase interaction, understanding and respect between sectors (Mikkelsen et al., 2013; van Vooren et al., 2020). Resolutions to procedural differences include shared personnel or resources, written agreements and holding regularly scheduled meetings made possible by policies, programmes and fundings that enable cross-sector partnerships (Ladekjær Larsen et al., 2020).

Parents in our study advocated for a ‘navigator’ (i.e. a facilitator or case manager or resource worker) to be the one contact for all aspects of care needed by the child. Research evidence supports collaborative care with a care manager to provide continuous support with the patients including initiating contact with services, follow-up and feedback, as well as facilitating patients to engage in self-management. Benefits to patients, the healthcare system and societal costs were reported to be higher for those with a case manager compared to usual care, for patients with depression (Holst et al., 2018). Navigator programmes have been used in care of chronic diseases such as cancer, diabetes, HIV, cardiovascular disease and dementia (McBrien et al., 2018). While navigator programmes appeared to improve processes of care, less is known about their impact with patient experience, clinical outcomes or costs.

A care ‘navigator’ would facilitate the continuum of care with a team approach and cross-sectoral collaboration as recommended by our study participants. A team approach encourages therapeutic engagement where children and their parents are listened to by building relationship with professionals and taking into account patients’ tolerance of treatment plan and proactive strategies (Bone et al., 2015). A multi-disciplinary team approach has been recommended in mental health services (Chafe and Audas, 2014); however, cross-sectoral collaboration amongst medical providers, social service workers, law enforcement and educators has yet to be explored in Canada. Children with complex mental healthcare needs often require services at the intersection of different sectors. Thus, future research is needed to determine how cross-sectoral corroboration would be managed to protect the privacy and confidentiality of the child and their families while also enabling information sharing to enhance care coordination.

### Strengths and limitations of study

A *strength* of this study is we had participation from across Alberta, including families from different socioeconomic levels and living in both rural and urban communities. Another strength is the insights provided by parents to the issues that have challenged them and the recommendations for strategies to address the gaps in the system which may be tested within the Alberta context and serve as a model for other jurisdictions.

The main *limitation* of this study is that despite our attempt to find families with both positive and negative experiences, the parents in our study mainly focused on the challenges and negative experiences. These families are more likely to participate in the study as they may be more inclined or invested to voice their concerns. Thus, our findings may be biased towards this singular experience, and less is known about families who have had a mostly positive experience with their services and the processes that worked well for them.

While we did not have enough data to develop into themes, a few parents did voice some of the positive aspects of their interactions within the healthcare system. For example, (1) one parent felt their doctor took time and listened to the parent’s concerns; thus, feeling heard and understood was important to the parent and the care they received; (2) another parent felt they had a voice in the decision-making process; (3) a third parent felt the practitioner acknowledged the parent as a person, was interested in their own mental health needs and self-care and inquired about the parent’s well-being; and (4) another parent said they appreciated the ability to provide input into aspects of treatment, such as duration choice to continue [the treatment] if effective or discontinue if not effective, and integrating different services and therapies, for example, medication, counselling, behavioural therapy and exercise. While some aspects of the patient–practitioner relationship were positive, navigating through the healthcare system seamlessly was the major challenge.

### Implications for practice

The findings of this study strengthen the current understanding of patient (i.e. children, parents and families) centred care for children with complex mental healthcare needs. Implications for practice inferred from the results of this study are multifarious. Firstly, an *implication for research* is the involvement of parents/families (as patient partners or consultants) in the discourse to address barriers and gaps in the healthcare system to find practical solutions in a timely and effective manner. As indicated in our study, the trauma experienced by parents navigating the system has not been well researched to inform changes to services and policy; thus, more research is needed to address this issue. Secondly, the *implications for practice* include creating a more centralized approach to assist parents/families to better navigate the system and enable parents/families co-design educational material to ensure better access of resources at community level. Thus, programme administrators and managers need to be given the ability (i.e. supported by policy) to create navigator positions and train frontline staff in making appropriate referrals in the pathway of services. Thirdly, the *implications for policy* are to impart multi-systems guidance for improving access to care, continuum of care across sectors of health, social services, education, among others providing services to children with complex mental healthcare needs to enable cross-sector collaboration. Thus, policies include (1) establishing model of integration of services by enabling coordination of the right service at the right time using a transdisciplinary approach, (2) creating effective lines of communication amongst healthcare providers and across-sectors to enable a continuum of care and (3) developing policies to support families and communities specifically addressing stigma by enabling cross-sector collaboration.

## Conclusion

This study identified the gaps and solutions to improve mental healthcare services in a Canadian context. For patients, it means their experience of care is connected and well-organized through their entire care journey, from care provider to care provider, from education to social services to health care and back again. To have an effective and supportive continuum of care, the healthcare system must communicate clearly to families. That is, patients would like to know (1) who is involved in providing and at times directing their care; (2) what is the plan of care and (3) what will happen next.

## Supplemental Material

sj-pdf-1-chc-10.1177_13674935211028694 – Supplemental Material for Working with parents of children with complex mental health issues to improve care: A qualitative inquiry Click here for additional data file. Supplemental Material, sj-pdf-1-chc-10.1177_13674935211028694 for Working with parents of children with complex mental health issues to improve care: A qualitative inquiry by Brenda MY Leung, Cynthia Wandler, Tamara Pringsheim and Maria J Santana in Journal of Child Health Care
