# Supplementary material for: Working with parents of children with complex mental health issues to improve care: A qualitative inquiry
Source: J Child Health Care. 2021 Jun 28;26(4):548–67. doi: 10.1177/13674935211028694 (PMC9667073; doi:10.1177/13674935211028694)
Supplement: sj-pdf-1-chc-10.1177_13674935211028694 – Supplemental Material for Working with parents of children with complex mental health issues to improve care: A qualitative inquiry [file sj-pdf-1-chc-10.1177_13674935211028694.pdf]

Appendix A – Interview Questions for semi-structured interview

A. Access & navigating mental health care

1. Think about your experience with getting care for your child; this may be getting the appropriate referral or ability to get to a particular clinic. What was that like?
2. Probes:
   - i. Ask about actual/estimate of distance from home to clinic. Has this posed any problems? Explain?
   - ii. Ask about transportation issues
   - iii. Ask about hours of operation

3. Think about the people who have provided care for you child. What has been your experience with family physicians? pediatricians? other specialist?

   Probe:

   a. Did you feel they understood your mental health concerns? Explain.

   b. Were you referred to other healthcare professionals? Explain.

   c. Was there a wait time to see specialists? Explain.

   d. Did you know what the wait time would be?

B. Use of healthcare and treatment options

This next set of questions is about any medical and non-medical treatments you have used for your child. These include:

- *Medical – family doctors, pediatricians, psychiatrists, etc.*
- *Allied health professionals – psychologists, counsellors, psychotherapists, etc.*
- *Other non-medical therapies – naturopaths, chiropractors, massage therapies, other therapists or therapies like herbs, vitamins/minerals, nutrients, etc.*

1.a. What types of treatments have been prescribed to your child?

[*Ask and list each treatment type by name, e.g. Medication #1, Medication #2, Psychotherapy, etc.*]

1.b. Go through each treatment, and ask the following:

i. Is your child still on this treatment? How long were they been on [*treatment #1*]

- If stopped, ask why did they stop?

ii. Who prescribed this treatment? (Ask about the type of practitioner)

iv. How do you feel about your child being on [*treatment #1*]?

v. Were you able to discuss concerns about the treatment with the prescriber?

- E.g. how long your child will continue to take [*treatment #1]*? Any plans for stopping [*treatment #1*]?

2. Did you feel engaged in decision making about the treatment your child was prescribed?

- Would you prefer a different level of engagement in making decision about the treatment your child receives?

3. Other than the treatments we've discussed, what other types have your child used?
   – ask and list as before.
   a. Please describe your experience with using [name the specific] therapies.
   b. How did you find out about these types of treatments?
   c. Why did you want to use this treatment for your child?

Probe specific therapeutic options:

Medical treatments

- Medication
- Psychiatry

Allied-health professionals

- Psychology
- Counseling

Other (non-medical) professions

- Naturopath/chiropractor
- Other practitioners (specify)

Natural Health Products (specify)
Dietary changes (specify)
Exercise (specify types)
Other activities (specify)

C. Searching for treatment options (facilitators & barriers)

1. At some point in your child's healthcare, did your child's healthcare needs change? Explain.
2. Tell me what it was like to look for services or information?
3. Have you looked for information about services or therapies outside the healthcare system?
   a. Where have you looked to find services/therapies outside the system?
